# Different Population Phenologies of *Grapholita molesta* (Busck) in Two Hosts and Two Nearby Regions in the NE of Spain

**DOI:** 10.3390/insects12070612

**Published:** 2021-07-06

**Authors:** Carles Amat, Dolors Bosch-Serra, Jesús Avilla, Lucía Adriana Escudero Colomar

**Affiliations:** 1 Department of Crop and Forest Sciences, University of Lleida (UdL), 25198 Lleida, Spain; carles.amatgomez@irta.cat; 2 Sustainable Plant Protection (Entomology), Institute for Agrifood Research and Technology (IRTA), Mas Badia, 17134 Girona, Spain; 3 Sustainable Plant Protection (Entomology), Institute for Agrifood Research and Technology (IRTA), 25198 Lleida, Spain; dolors.bosch@irta.cat; 4 Department of Crop and Forest Sciences, Agrotecnio-CERCA, University of Lleida (UdL), 25198 Lleida, Spain; jesus.avilla@udl.cat

**Keywords:** *Grapholita molesta*, population dynamics, phenology model, peach, apple, Spain, Catalonia

## Abstract

**Simple Summary:**

In two fruit-producing provinces in north-east Spain (Girona and Lleida), the moth pest (*Grapholita molesta*) can drastically affect production of their main fruit crops, apples and peaches, respectively. Populations of this pest can be monitored using traps; this enables determination of the optimal time to spray insecticides. This method requires labour and provides information about the population up to that date. Insect forecast models can complement this information, helping producers to anticipate increases in pest populations and thus allow more time to plan for moth control. However, informal observations indicate that the most used model in both regions does not predict precisely the evolution of the population of *G. molesta* in Girona. To start addressing this problem, we described the population dynamic of *G. molesta* and the commonly used prediction model was applied to both provinces. Significant differences were found between the provinces. In Girona, four generations were detected and five in Lleida; the length of each generation was higher in Girona. The prediction model did not fit the phenology of *G. molesta* in Girona, confirming that such models should be checked before applying them in regions other than the one for which they were originally created.

**Abstract:**

*Grapholita molesta* is an important pest of stone and pome fruits. In commercial orchards, integrated pest management programs use pheromone traps to monitor the population dynamics of *G. molesta* and adjust treatments. Phenology models can be used to forecast the population phenology of pests and to help optimise the time point at which to spray the orchards with insecticides. In the present study, the adult population phenologies of *G. molesta* in two provinces of north-east Spain were studied, as well as their fit to the phenology model most used in both provinces. Weekly captures of adults in pheromone traps through the season were recorded over 5 y in a large number of commercial orchards, and these data were used to determine the number of generations of *G. molesta* in each province. The results show significant differences between provinces in the generation time, being 97 degree days (DD) shorter in the Lleida province than in the Girona province. In Girona province, four generations were registered, while five were detected in Lleida. As a result of the differences found, the phenology model was not able to predict precisely the population dynamics in the Girona province.

## 1. Introduction

*Grapholita molesta* (Busck) (Lepidoptera: Tortricidae) is assumed to be a native of China, but it has spread over the world’s temperate areas following the expansion of its main host, the cultivated peach [1,2]. This pest is also known to occasionally attack pome fruits, mainly after nearby peach orchards have been harvested [2,3,4]. However, there are also reports of economic damage to apple orchards in Chile, Russia, Brazil, China, Italy, USA and Canada [5,6,7,8,9,10,11].

In Spain, *G. molesta* was first detected in 1974 in the Lleida province [12], where most peaches of the Catalonia Autonomous Community of Spain are cropped. In the early 2000s, *G. molesta* started to damage pome fruit orchards located in the Girona province (extreme NE of Spain) and also in Catalonia, where apples are the main crop fruit [13,14]. *G. molesta* have a high potential impact on these two provinces because most of the fruit production of Catalonia is concentrated in them. The fruits crops cultivated in these provinces involve several hosts of *G. molesta* (e.g., peaches, apples and pears) [15]. Several tools are available to control *G. molesta*: insecticide applications and mating disruption (MD) [16,17]. In the province of Girona, the attacks of *G. molesta* on apples made it necessary to install MD for *G. molesta* alongside MD for *Cydia pomonella* (L.), another key pest of apples from the moth family Tortricidae. In general, insecticide applications are used for both species to reinforce control with MD throughout the season, although control of *G. molesta* was not always enough to prevent damage [13,14].

*G. molesta* is a multivoltine species, presenting three to five generations depending on whether the environment is colder (e.g., Slovenia) or more temperate (e.g., California), respectively [18,19,20,21,22]. In mid-late autumn, when temperatures drop and the days shorten, the fifth instar larvae enter diapause until more favourable conditions occur at the end of winter [2]. Population dynamics and the annual number of generations of a target species are usually measured in field conditions by recording adult flights with pheromone-baited traps [18,20,23,24,25]. In the Girona and Lleida provinces, captures in traps and field observations indicate that *G. molesta* has at least four generations per year, but no studies have been conducted to confirm this.

Phenology forecast models are useful tools that predict the population dynamics of pests and can be used in IPM (Integrated Pest Management) programs to select the right moment to spray in order to control the target pest [26,27]. Phenology models can be tested through statistical and qualitative approaches, using, in the latter, graphical comparisons between observed and predicted data [28]. Several phenology models were developed for *G. molesta* (e.g., [18,23,29]). In the two provinces of the present study, the most used model to predict *G. molesta* progression in peach orchards is that of Croft et al. [18], currently included in the web site of the Department of Agriculture of the Catalan Government [30], because it was originally validated in both peach and apple orchards in the USA [18]. However, it was observed that in the Girona fruit-growing area, where apple is the main host of *G. molesta*, this phenology model is not precise enough to help in timing insecticide applications [13,14].

It is known that the development time for one species can vary between host species and even host varieties, as is the case in *G. molesta* [31,32,33,34,35]. The literature indicates that for *G. molesta*, on average, its development in apples requires between 20 and 60 more accumulated degree days (DD) than is required in peaches [33,34]. Therefore, more knowledge on the population dynamics, the number of generations per year and the biology of the pest within the conditions of the two provinces is needed to enable better fitting of the forecasting model. Consequently, the main goals in the present study are:

(a) to define the population dynamics of *G. molesta* in the Girona province where apple is the predominant fruit crop, as well as in the Lleida province where peach is the main fruit crop;
(b) to determine the number of generations of *G. molesta* per year in each province;
(c) to test the accuracy of the Croft et al. [18] phenology model for both provinces, including the development delay in apples reported by Myers et al. [33] and Yang et al. [34], with the final goal to know if the most used model in the study areas is useful or not for decision making in different crops and/or areas in which *G. molesta* is a problem.

## 2. Materials and Methods

### 2.1. Study Area

**Figure 1:**
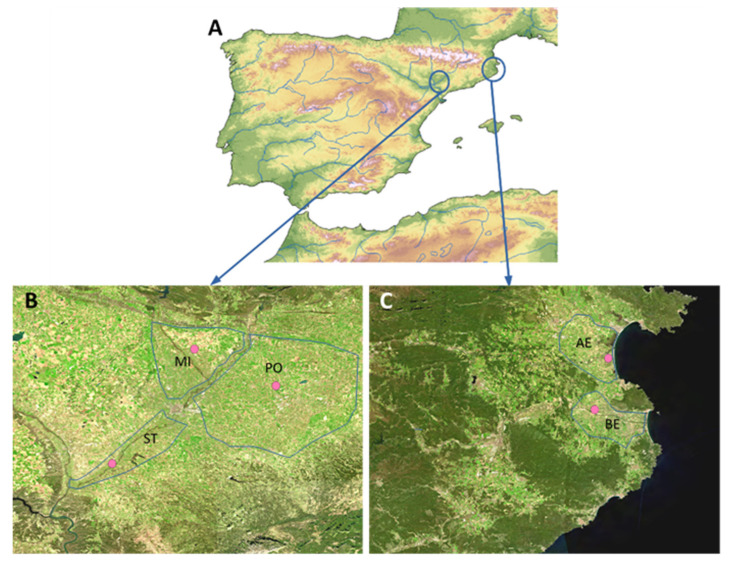
(**A**) Map of Spain showing the location of the two provinces of the north-east, (**B**) Lleida and (**C**) Girona, hosting the five fruit-growing areas studied: Girona: AE: Alt Empordà (pome fruit-growing area), BE: Baix Empordà (pome fruit-growing area), and Lleida: ST: Stone fruit-growing area, MI: Mixed stone and pome fruit-growing area and PO: Pome fruit-growing area. The locations of the official weather stations are indicated with pink dots.

Data used in this report come from commercial orchards of two provinces of the extreme north-east of Spain, Girona and Lleida, just 250 km apart (Figure 1A). The province of Girona has two main fruit-growing areas, in the north, the Alt Empordà, and the south, the Baix Empordà (Figure 1C). In both areas, apple is the main crop. Due to the fact that there are small climatic differences between both areas that translate into differences in the phenology of plants and pests, it was decided to keep both areas separated in studying the phenology of *G. molesta* and then fitting those data to the most commonly used forecasting phenology model in each case. In the province of Lleida, three areas were delimited based on the predominant tree cultivation: in the north, an area composed of mixed orchards of pome and stone fruit trees (hereafter Mixed area), in the south, an area with orchards mainly of stone fruit (hereafter Stone area), while in the east, a third area with predominance of pome fruit orchards (hereafter Pome area) (Figure 1B). The five areas studied share the same bioclimate and thermotype (related to a temperature range), but the two in the province of Girona differ from the three in the province of Lleida in ombrotype (related to the yearly rainfall), and also have slight differences in weather conditions (Table 1, [36]).

### 2.2. G. molesta Monitoring System

Data were collected from the monitoring white delta traps baited with Pherobank^®^ sex pheromone (Wijk bij Duurstede, The Netherlands), the standard used in all the studied areas. They were placed in commercial orchards in Girona province (apple orchards) and in Lleida province (peach orchards). Moth captures in traps were checked weekly from the beginning of March until the end of October. Data were collected during a 5-year study period (2015–2019) from orchards without MD. The number of traps varied between years in the five areas studied according to the number of orchards and traps that conformed to the study requirements: to use the monitoring material described above and not use MD to control *G. molesta* (Table 2). The remarkable decrease in the number of traps over time in the areas of Girona was due to the increasing number of apple orchards using MD to control the pest. Mean weekly captures and standard errors of those means were calculated for each area and year to draw the population dynamic curves.

### 2.3. Weather Recordings, G. molesta Phenology Model Fit and Degree Days Calculation

The daily maximum and minimum temperature were obtained from the official weather stations belonging to the Meteorological Service of Catalonia (Figure 1) [30]. The selected weather stations corresponded to the municipalities of Sant Pere Pescador for the Alt Empordà area, La Tallada d’Empordà for the Baix Empordà, Aitona for the Stone area, Albesa for the Mixed area and El Poal for the Pome area (Figure 1 and Table 1), because they were the most indicative of the weathers in each area studied. The model proposed by Croft et al. [18] and Rice et al. [23] predicts the first moth catch at 126 DD after January 1st and a generation time (from egg to egg) of 535 DD. The accumulated DD in each area was calculated from the daily temperatures using a single sine method of calculation described in Zalom et al. [37] with a lower threshold of 7.2 °C, an upper threshold of 32.2 °C and a horizontal cut off. Moreover, 60 DD (the maximum reported development delay when *G. molesta* larvae feed on apples compared to peaches [33,34]) was added to the number of DD required for the generation time in Girona areas to check whether this delay also fits to the development of *G. molesta* populations in apples. To assess the agreement between model predictions and the observed data at field, graphical plots were made. They were used to judge qualitatively the model’s ability to predict population dynamics [28].

### 2.4. Determination of the Beginning and the End of Each Generation

**Figure 2:**
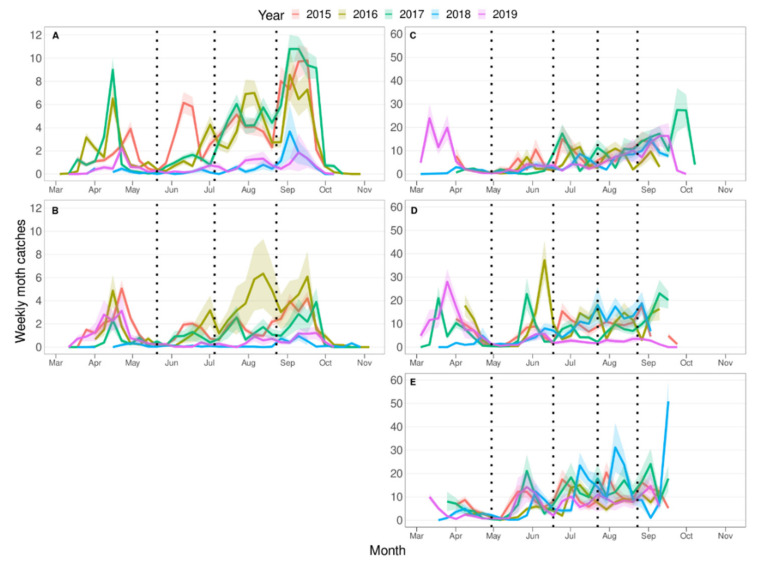
Weekly moth catches (mean ± SE) for each year (2015–2019) and for each area. (**A**) Alt Empordà and (**B**) Baix Empordà, both of Girona province; and (**C**) Stone area, (**D**) Mixed area, and (**E**) Pome area, the three of Lleida province. Dotted vertical lines indicate the approximate minima between flights. Shades of each line indicates the SE. Note: the *y*-axis scale is different for AB than for CDE.

Average weekly moth catches for each province and area were plotted for each year to determine the length of adult flight periods. The shift between two flight periods was determined from the minima between two flight period peaks. When no clear minima could be observed in any of the studied years due to overlaps between flights, this period was compared with the same period in the other years for the same area to find the most probable time point for when the shift occurs (for example, see the year transition between the third and fourth flight in year 2017; Figure 2A). The start of the first flight and the end of the last flight were only recorded in those years in which the data had a complete coverage of these specific periods. The duration of the flight was assumed to be the generation time from adult to adult and it was compared to the Croft et al. [18] model egg to egg generation time along with the corrected value when the larvae feed on apples. The accumulated DD was also used to follow and define each of the flights.

### 2.5. Statistical Analysis

Statistical analyses were performed with R version 4.0.3 (R Foundation for Statistical Computing, Vienna, Austria). A multiple-factor ANOVA (aov function) was conducted to determine the factors influencing the generation time expressed in DD. The factors analysed were: “province”, “area”, “observation year” and “seasonal flight order” (e.g., second flight). Stepwise model selection was used to remove those factors and interactions that were non-significant (*p* < 0.05). Normality was checked using the shapiro.test function and homoscedasticity with the bartlett.test. Tukey’s multiple comparisons test was calculated with the function HSD.test [38] for the factor “seasonal flight order” in each “province” separately. The standard error of the mean for the generation time was calculated using the function jackknife [39].

## 3. Results

### 3.1. Population Dynamics

Figure 2 shows the population dynamics of *G. molesta* throughout the season in the two fruit-growing areas of Girona province and in the three of Lleida province. The mean captures per week were lower in the Girona areas than in the Lleida areas. The maximum average number of captures in the Girona province (10 captures/trap/week) was registered in the Alt Empordà fruit-growing area in 2017, while in the Lleida province the maximum reached was 50 captures/trap/week in the Pome fruit area in 2018. The pattern of the population dynamics shows several differences between the provinces, e.g.: (i) *First moth capture*: In the two areas of Girona, the first moth was captured in mid-late March, while in the three Lleida areas, the first moth captured occurred in the second week of March; (ii) *First generation peak*: In the Girona province, the first generation peak was in mid-April and in the Lleida province it occurred in mid-March; (iii) *Generation identification*: In both provinces, the separation between the first and second generation was very clear, with 2–3 weeks of low captures, but from the second generation onward, in the Lleida areas, the separation between generations drastically decreased and some overlap appeared. Bimodal peaks were also present, which makes the separation between generations more complex, highlighting the value of a phenology model to identify each generation. In the two areas of the Girona province, it was easier to identify the different generations from the second generation onward. Nonetheless, to properly identify them, the phenology model was also applied; and (iv) *N ° of generation/season*: In Girona province, four generations were present in a year, while in Lleida province five generations occurred. Similarities between the provinces were also observed: (i) *Relative level of captures in each generation*: In general, the second generation had a lower number of catches than the first, and, from the third generation onward, the number of catches increased, the last generation being associated with the most catches; and (ii) *Timing of the final captures of the year*: Final captures were recorded at the end of September to the beginning of October in all the studied areas.

### 3.2. Phenology Model

**Figure 3:**
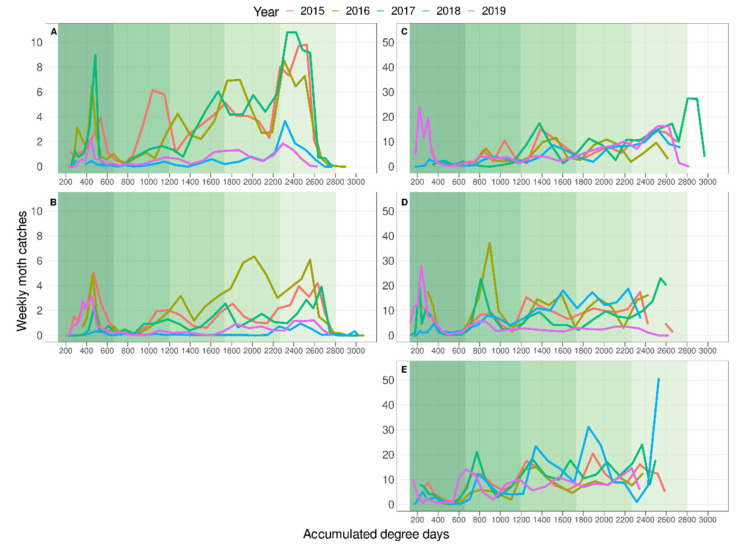
Mean weekly moth catches plotted against accumulated DD for each year (2015–2019) and for each area. (**A**) Alt Empordà and (**B**) Baix Empordà, both of Girona province; and (**C**) Stone area, (**D**) Mixed area and (**E**) Pome area, the three of the Lleida province. Background colours indicate the predicted generation range of the phenology model (535 DD) [18]. Note: the *y*-axis scale is different for AB than for CDE.

**Figure 4:**
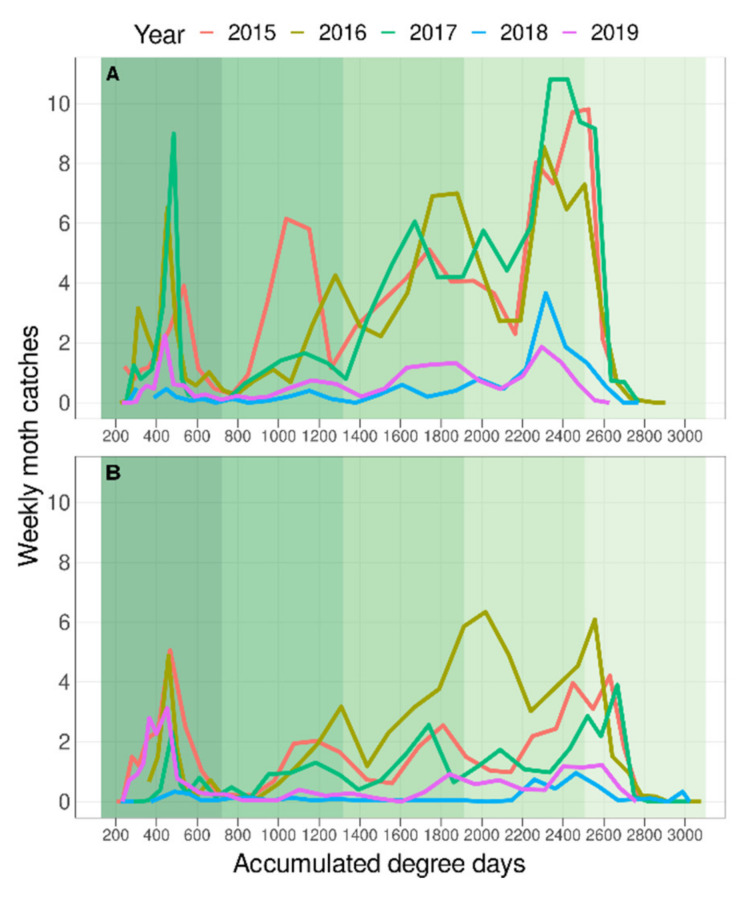
Mean weekly moth catches in the Girona province plotted against DD for each area and year. (**A**) Alt Empordà and (**B**) Baix Empordà, both of Girona province. Background colours indicate the predicted generation range of the phenology model [18] plus 60 DD for feeding on apples (595 DD in total) as reported by Myers et al. [33] and Yang et al. [34].

**Figure 5:**
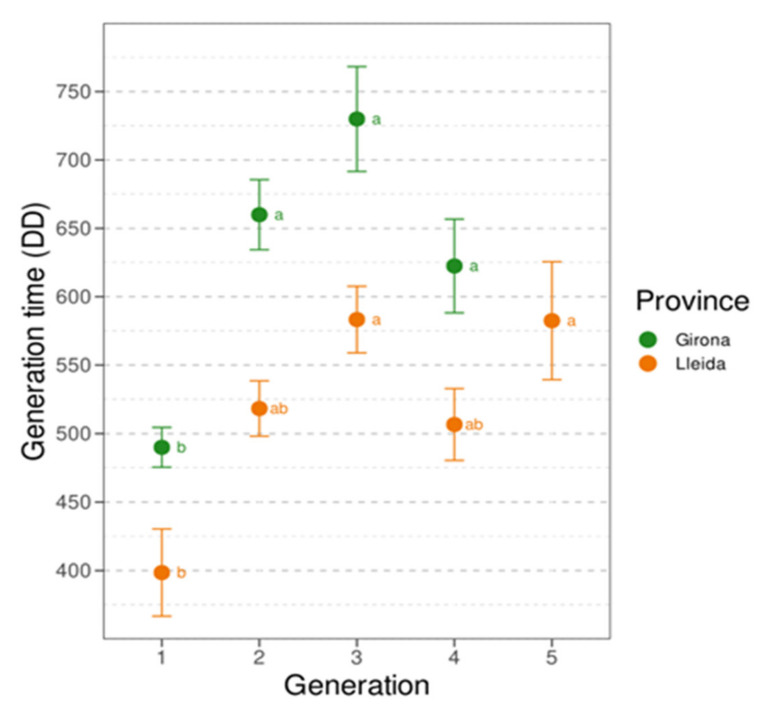
Duration of each generation in DD (mean ± SE) in each province. Different letters indicate significant differences between generations within a province (*p* < 0.05, Tukey).

The phenology model described by Croft et al. [18], applied to the weather data collected for each of the five areas, predicted five generations during the period when moths were flying in both the provinces studied. In the Girona province, after the second generation there was only partial correspondence between the predicted generations and those observed (Figure 3A,B, Table S1 of Supplementary Material), even after applying the maximum delay of 60 DD reported for *G. molesta* developing on apples [33,34] (Figure 4A,B, Table S1 of Supplementary Material). In the Lleida province, the phenology model accurately predicted all the generations in the Stone and Mixed areas (Figure 3C,D, Table S1 of Supplementary Material), but in the Pome area the generation peaks occur approximately 100 DD earlier than predicted (Figure 3E, Table S1 of Supplementary Material). Moreover, the first moth catch seems to be delayed in Girona while in Lleida it is close to the 160 DD predicted by the model developed by Croft et al. [18], although there are few data on this period in both provinces. The use of DD to represent the generations reduced the differences in flight synchronization between years within areas (Figure 3), and together with the representation of the phenology model helps to determine more accurately and easily each generation (compare Figure 2 and Figure 3 and Table S1 of Supplementary Material). All data analysed met the assumptions of normality and homoscedasticity (*p* > 0.05). There was a significant effect of the factors “province” (F_1,96_ = 10.17, *p* < 0.0001) and “seasonal flight order” (F_1,96_ = 38.7, *p* < 0.0001) on generation time. No differences were detected between “area” (F_3,92_ = 0.36, *p* = 0.784) and “year” (F_1,92_ = 0.57, *p* = 0.451) and no interactions among factors were detected. Regarding the generation time in each province, the results show that average generation time was higher in the Girona province (625.6 ± 2 DD) than in the Lleida one (528.1 ± 14.2 DD). The relative length of generations followed the same pattern in both provinces, but there were differences between the absolute generation length in each province. In both provinces, the first generation was the shortest and the third and fifth (a fifth generation only occurred in Lleida) were the longest (Figure 5). In the Girona province, the first generation was significantly shorter than all the others (see Table S2 in Supplementary Material). The Lleida first generation was also shorter than the third and the fifth, but it was not statistically different from the second and the fourth (see Table S2 in Supplementary Material).

## 4. Discussion

In this study, two main differences were found between the Girona and Lleida provinces, the first regarding the population dynamics of *G. molesta* and the second in the fit to the phenology model currently used to forecast population progression over a season. The main difference is that in the Girona province, four generations were observed, while in the Lleida province there were five, despite moths being captured for a similar time period in both regions. The population dynamics observed in both provinces follows the same pattern already reported in other studies based on experimental or untreated orchards [19,24,29,40]. Ellis [41] suggested that the use of moth capture data from orchards treated with insecticide applications makes population dynamics analysis harder. In the present study, although data were obtained in commercial orchards without MD and insecticides were used to control pests, similar population dynamics curves were obtained to those previously reported for other areas of the world for untreated experimental orchards. Therefore, analysis of data coming from a high number of orchards may have reduced the influence of the use of insecticides on the population dynamics curves. Nevertheless, other authors have demonstrated that it is possible to validate a phenology model using data obtained in orchards with insecticide applications [42]. 

The number of captures was generally lower in the Girona province than in the Lleida province. This difference may be due to a lower survival of *G. molesta* in apples [43], although specific studies for the populations of the study area are lacking. Other factors that can influence the lower moth capture rates in the Girona province are the high number of orchards using MD for *G. molesta* as a control method and the absence of stone fruit orchards. The effect of MD in reducing moth population levels when MD is applied in a large area is well known [17,44,45,46]. The highest number of captures was found in the Lleida province in the Pome area, at the end of the flying period. Even though apples predominate in this area, peaches are also grown. Only occasional economic damage has been reported on late-growing varieties of apples in this region; therefore, MD against *G. molesta* is only applied to the isolated peach orchards cropped between the apple orchards, reducing the effect of MD and allowing migration of nearby mated females from untreated zones once most of the peach orchards have been harvested [45,47].

The first moth is captured later in the Girona province than in the Lleida province. This fact is not only reflected in the flight start date but also in the fact that in Girona, *G. molesta* requires more accumulated DD for the flight start. However, the exact moment when the moths begin to fly is still unknown, since in some years, and especially in the three areas studied in the province of Lleida, the phenology model predicted the beginning of the first flight before the monitoring traps were placed in the area. In addition to this, in some years, the first portion of the moth catch curve is descending, suggesting that the first flight was already present when the first monitoring traps were set. Higher temperatures during diapause development can lead to a lower development rate [48]. Because first moth catches begin earlier in Lleida, even though winter temperatures there were lower than in Girona (Table 1), our results suggest that the optimum temperature for diapause development in *G. molesta* seems close to the temperatures registered in Lleida. However, diapause termination is controlled by many factors in *G. molesta*, and some of them are still unknown [2,40,48]. Therefore, more studies on the diapause of this pest in both provinces will help in better understanding the differences observed between areas and in turn improving the precision of the phenology model, with regard to the first generation. Another factor that can have an influence on the timing of the first moth catch is the crepuscular temperature. The flight of male *G. molesta* is halted when temperatures are below 15 °C, and in early spring moths become caught in pheromone traps between 4 and 1 h before sunset [49,50]. During the period when the first moths appear in both provinces (mid-February to mid-April), the average daily temperature of the five areas over the five years of the study was below the reported threshold or slightly above (considering the standard deviation) (Alt Empordà: 12.7 ± 2.7 °C, Baix Empordà: 13.0 ± 2.8 °C, Stone area: 14.7 ± 3.8 °C, Mixed area: 13.1 ± 3.6 °C, Pome area: 13.2 ± 3.6 °C, [30]). This may therefore influence the timing of the first moth catches.

In contrast, the final captures in the season happen at a similar time of the year in both provinces (early October). Photoperiod is a key factor regulating the start of diapause in *G. molesta* [2,40,51] and, in the present study, both provinces had a very similar photoperiod (maximum 3 min difference between provinces; [52]).

Differences in the generation time of *G. molesta* in successive generations were reported by Damos and Savapolou-Soultani [29], and we report similar findings in the present study. One of the factors that can influence the development of the pest is the photoperiod, especially in the last generations when the photophase is shorter than that of the generations developed during summer [51]. Bimodal peaks and generation overlaps were also reported for this species [29,40]. These two factors make the mathematical validation of a phenology model difficult in this species. For this reason, and to check whether or not the model is useful in predicting the phenology of the insect, the approach proposed in this study was adopted. Both characteristics appear more conspicuously in the populations in the Lleida province than in Girona. Damos and Savapoulou-Soultani [29] suggested that extreme high temperatures might explain the overlapping of generations. Given that in Lleida the daily thermal amplitude during the flying period of *G. molesta* is higher than in Girona (Table 1), our results support this. In addition, there is a greater mixture of host crops in the Lleida province, which may contribute to there being more overlapping of generations due to different development rates of moths in different hosts and varieties [31,32,33,34,35]. The use of DD to represent the population dynamics, instead of date, reduces the effect of temperature over the years and helps to identify generations, despite overlaps, as demonstrated in the present study (as an example, refer to year 2016, third and fourth generations, in Figure 2C and Figure 3C).

Several studies recommend evaluating phenology models in the local conditions of the area of interest due to possible adaptations of the pest [53,54,55,56]. Even though the model described in Croft et al. [18] was validated in the USA (California and Michigan), on peach and apple orchards (generation time: 535 DD), the results obtained in the present study confirm the need to evaluate phenology models in local conditions. While the Lleida population has a similar generation time to the USA (528.1 ± 14.2 DD), in Girona the development time is much higher (625.6 ± 2 DD) despite the fact that the two provinces are separated by only 250 km. The difference between the generation time predicted by the model and the real generation time registered in the province of Girona correspond to the DD accumulated in 2 or 3 days (depending on the temperature registered during the development period of each generation). These 2 or 3 more days are a small error in the model when predicting the phenology of the pest, but it accumulates with each generation [38], resulting in an error large enough to reduce the efficacy of most insecticide applications due to lack of timing between the phenology of insects and the insecticide application. Due to the fact that the second, third and fourth generations are longer in Girona province than that predicted by the phenology model, the error in the prediction of these generations would be around 12–15 days. The delay in the development of the pest on apples [38,39] could partially explain the increase in the DD needed in the Girona areas, although the DD increase found in the present study corresponds to 2.3 times more than if the DD was the maximum of 60 reported in the literature. Differences in development time within apple varieties was also observed [31,33,35], so more information about the time of development of the species in the varieties cultivated in the studied areas should help to better understand the results obtained and to improve the phenology model.

On the other hand, in the province of Girona, the delay in the start of the first generation added to the increase in the duration of the following generations, suggest an adaptation to local conditions [25]. This adaptation could also be a result of an increase in the lower development threshold temperature in populations of Girona, as it was reported in other areas of the world [22]. Although the two provinces share the same bioclimate, it seems that the small differences in some climatic conditions (e.g., those corresponding to the ombrotype) as well as the different hosts present in the two provinces, could have induced/facilitated the development of local adaptations in the populations. This hypothesis requires study to develop a phenology model that effectively forecasts the development of the *G. molesta* population throughout the year when the species develops in apples in the Girona province.

Most papers on phenology model validation use data from a few orchards (i.e., [18,19,22,25,26,29,42,55,56]). This document uses a large data set from two provinces and for a period of 5 years for phenology model validation. Although some data obtained with the model were used here to evaluate the same model, the potential negative effects of this approach were minimized by using a multi-year field data set in addition to visual validation. In Lleida province, a good fit of the predicted phenology by the model to the population dynamics of *G. molesta* was found, while in Girona the model did not properly predict the development of the pest. These results are practical and useful because they explain the difficulties found at the field level in predicting the development of the pest based on the model and, therefore, in deciding the appropriate time to spray. These results also show the need for an adjustment of the model used, or for development of a new one, in order to facilitate decision making at the field level.

## 5. Conclusions

In our study, we found a different population phenology of *G. molesta* in two closely located fruit-producing provinces growing different crops. These differences result in four generations of the moth a year in the Girona province and five in the Lleida province. The phenology model commonly used in both provinces, developed by Croft et al. [18], does not predict accurately the population dynamics of the species in the Girona province where apples are the main crop, but it has an acceptable fit to the data for the Lleida province, where peaches are the main crop, including in the areas with a mix of peach and pome fruits. As a consequence, in the Girona province it cannot be used as a decision-making tool to control *G. molesta*.

Developing phenology models for a multivoltine species over large areas requires in-depth knowledge and understanding of the biology of the species in the environments and crops in which it grows. Thus, it seems clear that studies on the development of *G. molesta* on apple and peach are necessary, to better fit a model for each of the provinces considered in this document. This will allow us to identify the differences in biology of *G. molesta* that explain the different population dynamics found between the provinces, and will open the way to adjust a model to the development of the species in each one.

Finally, the methodology used in the present document was shown to be practical for making faster decisions regarding the proper fit of a phenology model in a new area or the need to adjust it. 

## Figures and Tables

**Table 1:** Bioclimatic (based on the Worldwide Bioclimatic Classification System) and meteorological data for the five studied areas. Meteorological data were obtained from the official weather stations associated with each area from 2015–2019. The UTM (Universal Transverse Mercator) coordinates for each weather station are indicated. Minimum and maximum temperatures for the period of diapause of *G. molesta* were calculated from October to February, and equivalent temperatures for the developing period from March to September.

Area	Bioclimate		Bioclimate Belt		Weather Data					
	Type	Subtype	Thermotype	Ombrotype	Station Location	Min. Diapause Temp.	Max. Diapause Temp.	Min. Devel. Temp.	Max. Devel. Temp.	Yearly Rainfall
Alt Empordà, (Girona)	Mediterranean pluviseasonal oceanic		meso-Mediterranean	subhumid	507995X 4669451Y (31T)	4.80	16.85	12.38	24.49	546.98
Baix Empordà (Girona)	Mediterranean pluviseasonal oceanic		meso-Mediterranean	subhumid	505127X 4655771Y (31T)	4.51	16.84	12.48	25.28	541.84
Stone area, (Lleida)	Mediterranean pluviseasonal oceanic	steppic	meso-Mediterranean	dry	288002X 4595926Y (31T)	3.30	15.47	11.41	28.09	315.46
Mixed area, (Lleida)	Mediterranean pluviseasonal oceanic	step-pic	meso-Mediterranean	dry	306325X 4625793Y (31T)	3.47	13.77	11.85	26.52	382.68
Pome area, (Lleida)	Mediterranean pluviseasonal oceanic		meso-Mediterranean	dry	323310X 4615624Y (31T)	2.37	13.96	10.95	26.68	350.36

**Table 2:** Number of traps fulfilling the study requirements in each area and year. AE: Alt Empordà, BE: Baix Empordà, ST: Stone area, MI: Mixed area, PO: Pome area.

Province	Area	Number of Traps				
		2015	2016	2017	2018	2019
Girona	AE	104	109	111	15	40
	BE	167	78	53	23	25
Lleida	ST	17	37	13	52	30
	MI	47	32	25	13	23
	PO	32	52	14	21	16

## Data Availability

The data presented in this study are available on request from the corresponding author.

## Supplementary Materials

The following are available online at https://www.mdpi.com/article/10.3390/insects12070612/s1. Table S1: DD predicted and registered; Table S2: Mean pairwise comparison of the different generation times.
