# Supplementary material for: Different Population Phenologies of Grapholita molesta (Busck) in Two Hosts and Two Nearby Regions in the NE of Spain
Source: Insects. 2021 Jul 6;12(7):612. doi: 10.3390/insects12070612 (PMC8307000; doi:10.3390/insects12070612)
Supplement: Supplementary file 1 [file insects-12-00612-s001.zip › insects-1282047-supplementary.pdf]

Table S1. DD predicted by the phenology model (Croft et al. [18]), applying the correction of 60 DD delay (Myers et al. [32] and Yang et al. [33]) and the registered in each area in each of the years studied.

Moth catches	Predicted by phenology model (535DD)	Predicted by phenology model (595DD)	DD registered up to the event																								
			Girona										Lleida														
			Alt Empordà					Baix Empordà					Stone area					Mixed area					Pome area				
			2015	2016	2017	2018	2019	2015	2016	2017	2018	2019	2015	2016	2017	2018	2019	2015	2016	2017	2018	2019	2015	2016	2017	2018	2019
First moth catch in the season	126	126	250	250	250	250	300	250	350	350	350	250	*	*	300	200	150	*	*	150	200	100	*	*	*	160	*
Minima in between 1st and 2nd generation	661	721	750	800	700	750	750	800	800	800	800	800	600	625	550	700	550	550	550	550	550	550	575	550	500	600	500
Minima in between 2nd and 3rd generation	1196	1361	1250	1500	1350	1400	1400	1550	1450	1400	1450	1600	1250	1150	900	1250	1175	1125	1100	1050	1100	1000	1025	1100	975	1150	925
Minima in between 3rd and 4th generation	1731	1911	2175	2150	2125	2100	2100	2150	2250	2300	2150	2150	1800	1775	1600	1900	1575	1650	1725	1750	1750	1600	1650	1700	1550	1700	1300
Minima in between 4th and 5th generation	2266	2506	2725	2800	2775	2700	2550	2850	2900	2825	3000	2750	2175	2250	2100	2275	2325	2100	2150	2250	2350	2000	2150	2250	2150	2300	1800
Final 5th generation	2801	3101	-	-	-	-	-	-	-	-	-	-	2850	2650	3000	2850	2850	2700	*	2750	*	2550	2650	*	*	*	2400

(-) = No flight produced; (*) No enough data for the event determination

Table S2. Mean pairwise comparison of the difference generation times of *G. molesta* (in DD) in two generations of the same year, for each province. p-values were calculated with a Tukey test. Significance codes: '.' 1-0.05, '*' 0.05-0.01, '**' 0.01-0.001, '***' <0.001.

Province	Generations compared	Generation time difference (DD)	p-value	
**Girona**	1-2	-170.00	0.0014	**
	1-3	-240.00	0.0000	***
	1-4	-132.50	0.0160	*
	2-3	-70.00	0.3534	
	2-4	37.50	0.8073	
	3-4	107.50	0.0665	.
Lleida	1-2	-119.76	0.0744	.
	1-3	-184.76	0.0012	**
	1-4	-108.10	0.1320	
	1-5	-183.93	0.0033	**
	2-3	-65.00	0.3813	
	2-4	11.67	0.9976	
	2-5	-64.17	0.5080	
	3-4	76.67	0.2226	
	3-5	0.83	1.0000	
	4-5	-75.83	0.3383	
